# A latent profile analysis of positive psychotic symptoms and dissociative symptoms in the general population: their associations with childhood trauma and outcomes

**DOI:** 10.1007/s00127-025-02992-3

**Published:** 2025-09-15

**Authors:** Bryan Ho-wang Yu, Anson Kai Chun Chau, Chui- De Chiu, Suzanne Ho- wai So

**Affiliations:** Department of Psychology, The Chinese University, 3/F Wong Foo Yuan Building, Hong Kong SAR, China

**Keywords:** Psychotic-like experiences, Psychosis, Dissociation, Childhood trauma, Latent profile

## Abstract

**Purpose:**

Psychosis and dissociation are inter-correlated syndromes. As they are both multifaceted constructs, individuals experiencing positive psychotic and dissociative symptoms may have heterogeneous presentations of co-occurring symptomatology. This study aimed to identify phenotypes of individuals with varying degrees of these co-occurring symptoms in the general population, while also examining the impact of childhood trauma and prospective emotional and functional outcomes associated with these phenotypes.

**Method:**

Participants were recruited from the general population through diverse means. At baseline and six months after, adults (age 18–65) were assessed for positive psychotic symptoms, dissociative symptoms, depression, anxiety, and functioning using an online survey. Childhood trauma was assessed at baseline only. Distinct subgroups were estimated by latent profile analysis, with childhood trauma examined as predictor of the profiles. The identified profiles were compared on emotional and functional outcomes at both timepoints.

**Results:**

The community sample consisted of 2,958 individuals (mean age = 34.69; 72.5% female). Four distinct profiles were identified – ‘low overall’, ‘moderate (hallucinatory)’, ‘moderate (dissociative)’, and ‘high overall’. Emotional/sexual abuse and physical neglect notably differentiated the ‘low overall’ profile from the other three profiles, with sexual abuse specifically linked to the ‘high overall’ and ‘moderate (hallucinatory)’ profiles. The ‘high overall’ and ‘moderate (dissociative)’ profiles exhibited persistent elevated depression across timepoints than the other profiles.

**Conclusions:**

Distinct profiles of varying levels of co-occurring positive psychotic and dissociative symptoms were identified in the general population. The implications for early identification and intervention of these commonly co-occurring symptoms are discussed.

**Supplementary Information:**

The online version contains supplementary material available at 10.1007/s00127-025-02992-3.

## Introduction

While psychotic disorders and dissociative disorders are distinct mental health conditions with clearly defined diagnostic criteria, positive psychotic symptoms and dissociative symptoms commonly co-occur within these two groups of patients [1–3]. In patients with psychotic disorders, co-occurring dissociative symptoms (such as depersonalisation/derealisation, and amnesia) are associated with more severe psychosis and worse clinical outcomes [4, 5]. In patients with dissociative disorders, having co-occurring or past positive psychotic symptoms (such as delusions and hallucinations) are also associated with more severe symptomatology, and more self-harm and suicidal attempts [6].

Positive psychotic symptoms and dissociative symptoms also occur in individuals without psychiatric diagnoses, implicating a continuum of the psychopathologies across clinical and non-clinical groups [7, 8]. Cross-sectionally, non-patients with higher positive psychotic and/or dissociative symptoms often exhibit higher levels of distress and functional impairment and may show tendencies towards self-harm and suicide compared to those with fewer or no symptoms [9, 10]. Longitudinal research has also shown that individuals with higher positive psychotic or dissociative symptoms at baseline face an elevated risk of developing other mental health problems [11, 12], including a full-blown psychotic disorder [13]. Analogous to the clinical samples, positive psychotic symptoms and dissociative symptoms have been shown to be inter-correlated in community samples [14–17].

While there is an increase in interest in the relationship between positive psychotic symptoms and dissociative symptoms beyond individuals with established diagnoses, this area of research can be further advanced in several ways. Firstly, while many existing studies utilised small and unrepresentative (e.g., student) samples only (e.g [14, 15]), an inclusion of wider and more representative samples from the general population will improve generalisability of results. Secondly, most studies measured symptoms at a single time point only, whereas incorporating repeated measures of symptoms would provide insights into their relationships with emotional and functional outcomes, such as risks of mood and anxiety disorders, and poorer social and occupational engagement [18, 19].

Thirdly, most studies either regarded the symptoms as singular constructs, or focused on a limited range of symptoms only (especially for dissociative symptoms [20, 21]). Contemporary conceptualisations of positive psychotic symptoms consist of various facets such as delusions, hallucinations, disorganisation [22]; likewise, dissociative symptoms include depersonalisation/derealisation, memory disturbances, emotional numbness, identity confusion and alteration [23, 24]. In view of the heterogeneity within either set of symptomatology [22, 25], analysing the diverse symptom dimensions as separable constructs rather than collapsing them into total score of psychosis/dissociation respectively would deliver more comprehensive insights into how the two symptom continua manifest and interact with each other within the general population.

Lastly, this area of research would benefit from a more sophisticated approach to measuring symptom occurrence and co-occurrence. Previous studies have either tested the correlations between positive psychotic symptoms and dissociative symptoms (for review [26]), or divided individuals into high/low scorers on one symptom and then compared the subgroups on the other symptom (e.g [17]). While these approaches could confirm a positive association between levels of positive psychotic symptoms and dissociative symptoms, latent profile analysis (LPA) could extend the investigation by discovering subgroups of individuals characterised by differential symptom profiles (e.g [9, 27]). Based on previous correlational studies, it is anticipated that at least one profile characterised by co-occurring positive psychotic and dissociative symptoms will emerge, whereas other profiles may score high on either or neither set of symptoms.

LPA also enables the identification of potential risk factors contributing to one or both of these symptom sets while simultaneously estimating the profiles. Childhood trauma is an established risk factor for psychosis and dissociation respectively [28, 29]. Converging evidence supports a dose-response relationship between childhood trauma and psychotic/dissociative symptoms [30, 31]. On the other hand, specific *types* of childhood trauma are proposed to impact symptoms differentially. Previous studies have reported certain subtypes (e.g., physical, sexual abuse) to be preferentially associated with positive psychotic symptoms or dissociative symptoms [32–34], whereas others found no evidence for specificity [35]. The specificity account hence remains to be clarified.

The aim of the current study was threefold: (1) to identify profiles of positive psychotic and dissociative symptoms in a large community sample; (2) to examine the contribution of childhood trauma in predicting these profiles; and (3) to compare the profiles on emotional and functional outcomes cross-sectionally and prospectively (over six months).

Key hypotheses were:

(i) Multiple profiles of individuals presenting with varying degrees of positive psychotic symptoms and/or dissociative symptoms will be revealed by latent profile analysis.
(ii) Cumulative childhood trauma, potentially child abuse subtypes, will predict profiles with high scores on both positive psychotic and dissociative symptoms, followed by those who are high on either set of symptoms, and then those who are low on both.
(iii) Emotional and functional outcomes at baseline and six months will be worst among profiles with high scores on both positive psychotic and dissociative symptoms, followed by those who are high on either set of symptoms, and then those who are low on both.

## Method

### Participants

Hong Kong residents aged 18–65 who can read Chinese were recruited from the general population, through mass emailing in local universities, snowball sampling, Facebook (through paid advertisement to randomly reach users according to the inclusion criteria), and distribution of pamphlets around local communities. With an intention to capture the full range of symptomatology in the general population, no exclusion criteria were adopted.

According to Wang et al. [36], an LPA with 8 indicators (3 positive psychotic symptom dimensions, 5 dissociative symptom dimensions; see Measures below) would yield a good entropy value (classification accuracy) of 0.80 to 0.84 when the sample size is between 1000 and 3000 [36]. In view of the published prevalence estimates for positive psychotic and dissociative symptoms (7.2% and 3.4% respectively [8, 10]), and assuming 20% invalid responses and a 30% attrition rate, approximately 3,000 participants would ensure that even the smallest profile would have at least 57 valid responses at baseline (3000 × 3.4% x [1–20%] x [1–30%] = 57.12), which is higher than the recommended minimum of 25 participants per profile [37].

## Measures

### Indicator variables

**Positive psychotic symptoms**. Positive psychotic symptoms were assessed by the 15-item Community Assessment of Psychic Experiences-Positive Scale (CAPE-P15; [38]), a brief version of the CAPE [39]. CAPE-P15 assesses three dimensions of positive psychotic symptoms: persecutory ideation, bizarre experiences, and perceptual abnormalities. Frequency of each item is rated on a 4-point scale (1, ‘never’ to 4, ‘nearly always’). A total frequency score above 1.47 suggests an ultra-high-risk state for psychosis (sensitivity = 77%, specificity = 58%; [40]).

**Dissociative symptoms**. Dissociative symptoms were measured by the 30-item Dissociative Experience Measures Oxford (DEMO; [20]). The scale measures five dimensions of dissociative symptoms: unreality, numbness/disconnectedness, memory blanks, zone-out, and vivid internal world. Each item is rated on a 5-point scale from 1 (‘not at all’) to 5 (‘most of the time’).

## Predictor variable

**Childhood trauma.** Twenty-five items of the Childhood Trauma Questionnaire-Short Form (CTQ-SF; [41]) assessing child abuse (physical, emotional, sexual) and neglect (physical, emotional) were rated on a 5-point scale from 1 (‘never true’) to 5 (‘always true’). The presence of each trauma was determined with a binomial score, based on Bernstein and Fink’s cut-off [42] for ‘at least moderate to severe’: physical abuse (≥ 10), emotional abuse (≥ 13), sexual abuse (≥ 8), physical neglect (≥ 10), emotional neglect (≥ 15). The scores were then summed to produce a cumulative trauma score ranging from 1 to 5.

## Outcome variables

**Mood symptoms.** Patient Health Questionnaire-9 (PHQ-9; [43]) and the General Anxiety Disorder 7-item Scale (GAD-7; [44]) were used to measure levels of depression and anxiety in the past two weeks respectively. For both scales, each item is rated on a 4-point scale from 0 (‘not at all’) to 3 (‘nearly every day’).

**Daily Functioning.** Functional impairment was assessed by the 12-item version of the World Health Organisation Disability Assessment Schedule 2.0 (WHODAS-2.0; [45]) on a 5-point scale from 1 (‘none’) to 5 (‘severe’). The normative mean for the general population was 3.1 [46].

### Other variable

As DEMO is a newly developed scale and its application in diagnosable cut-off remains untested, a total score of 20 or above of the Dissociative Experiences Scale-Taxon (DES-T; [47]) was used as a reference cut-off for a potential dissociative disorder.

## Procedure

At baseline, participants filled out an online survey covering basic demographics and the measures mentioned. At 6-month follow-up, survey completers at baseline were invited to complete the same measures for indicator and outcome variables. Each participant received USD 8.90 for participation at each time point.

### Data analysis

Response validity was assessed according to Curran’s suggested criteria [48]. Responses were excluded from analysis if they (1) were duplicates (based on participant name and contact details); (2) failed three out of five attention check items (e.g., ‘Please choose “4” for this question’); and (3) had a completion time below the product of number of items×2s [49]. Because a forced-choice setting was used in the survey, all items were completed and there were no missing data in the submitted responses. Multivariate outliers indicated by Mahalanobis distance with *p* <.001 were further removed [37].

Descriptive statistics of the variables and the LPA were computed using IBM SPSS Statistics (Version 27) and Mplus Version 8 [50] respectively. Key variables were transformed into z-scores before entering into LPA. Each individual symptoms of CAPE-P15 and DEMO were entered as indicator variables. Given a non-normal distribution of the indicator variables, latent profile models were estimated using the maximum likelihood estimation with robust standard errors [37], with 500 initial stage random starts and 20 final stage optimisations.

Models of two to six profiles were estimated and compared based on the following criteria of model fit: Akaike information criteria (AIC), Bayesian information criteria (BIC), sample size adjusted BIC (ssBIC), and entropy. The relative improvement of model fit between *K*-profile and *K*−1 profile models was evaluated by the bootstrapped parametric likelihood ratio test (BLRT) using 20 samples, 100 draws, and 200 random starts. For comparisons of key variables across profiles, chi-squared tests and ANOVA/MANOVA were used for categorical and continuous variables respectively. 

Hypothesis 2 was examined through multinomial logistic regression using Vermunt’s three-step approach [51], with latent profiles as dependent variables and childhood trauma variables as independent variables. To test Hypothesis 3, mean level of outcome variables at baseline and follow-up were compared across profiles using the BHC three-step approach [52].

## Results

### Sample characteristics

At baseline, 4,135 participants responded to the survey, among whom 3,206 (77.5%) completed all the items. Two-hundred-and-one participants failed the validity check and were excluded (attention check items: *n* = 90; duplicates: *n* = 53; completion time: *n =* 58). Forty-seven outliers were removed. The final sample consisted of 2,958 participants (92.3%), with a mean age of 34.69 years (SD = 12.45) and the majority being female (*n* = 2146, 72.5%). Detailed demographic characteristics of the final sample and descriptive statistics of key variables (mean, standard deviations, Cronbach’s α) are presented in Supplemental Table 1.

### Latent profile analysis

Correlations of indicator variables at each time point are shown in Supplemental Table 2. All correlations were significant (Spearman’s rho = 0.18–0.68, *ps* < 0.001). Table 1 presents fit indices for each model. The best log-likelihood values were replicated for two- to four-profile models only. All model-fit indices and the Bootstrapped LRTs supported the superiority of the 4-profile model. Considering the model goodness-of-fit, model parsimony, and conceptual interpretability, a 4-profile model was selected as the optimal solution.

**Table 1:** Model fit indexes for latent profile models

	LL	AIC	BIC	ssBIC	Entropy	Bootstrapped LRT
1-Profile	−29106.191	58300.38	58564.04	58424.24	NA	N/A
2-Profile	−27902.020	55910.04	56227.63	56059.23	1.000	< 0.001
3-Profile	−27143.514	54411.03	54782.55	54585.55	0.999	< 0.001
4-Profile	−26851.113	53844.23	54269.68	54044.08	0.962	< 0.001
5-Profile	−25757.262	51674.52	52153.91	51899.72	0.966	Could not compute
6-Profile	−24504.770	49187.54	49720.85	49438.07	0.970	Could not compute

Note. AIC = Akaike Information Criterion; BIC = Bayesian Information Criterion; LL = log-likelihood; LRT = likelihood ratio test; ssBIC = Sample Size Adjusted BIC. The LRT could not be computed for 5- and 6-Profile models because the best log-likelihood values were not replicated

**Table 2:** *Descriptive statistics of symptoms and childhood trauma within identified profiles (N = 2*,*958)*

	Profile				Post-hoc tests (Bonferroni)
	1	2	3	4	
No. of individuals, % (*n*)	77.2% (2284)	12.8% (379)	7.5% (223)	2.4% (72)	
No. of individuals with lifetime psychiatric diagnosis, % (*n*)	1.6% (37)	3.4% (13)	2.2% (5)	5.6% (4)	
	**Mean (SD)**	**Mean (SD)**	**Mean (SD)**	**Mean (SD)**	
**Age**	35.31 (12.56)	34.02 (12.16)	30.34 (11.25)	32.17 (11.11)	2 > 3; 1 > 3
**Gender**					
Female, % (*n*)	72.9% (1665)	70.7% (268)	75.8% (169)	61.1% (44)	n.s.
Male, % (*n*)	27.1% (619)	29.3% (111)	24.2% (54)	38.9% (28)	
**CAPE-P15**					
Total score	20.15 (3.48)	24.75 (4.32)	23.87 (4.71)	30.56 (4.35)	4 > 2 > 3 > 1
Persecutory ideation	8.03 (1.93)	9.33 (2.23)	9.61 (2.47)	10.10 (2.06)	4 > 2 > 1; 3 > 1
Bizarre experiences	9.12 (2.15)	11.12 (2.73)	11.25 (2.84)	13.90 (2.78)	4 > 3 = 2 > 1
Perceptual abnormalities	3.00 (0.00)	4.30 (0.46)	3.01 (0.12)	6.56 (1.01)	4 > 2 > 3 = 1
**DEMO**					
Total score	45.41 (11.42)	59.80 (15.21)	69.42 (13.44)	75.47 (14.08)	4 > 3 > 2 > 1
Unreality	7.38(1.94)	10.52 (3.76)	16.23 (2.93)	13.90 (4.57)	3 > 4 > 2 > 1
Numbness/disconnectedness	10.09 (4.14)	13.06 (4.75)	15.50 (4.74)	15.94 (4.68)	4 = 3 > 2 > 1
Memory blanks	7.53 (2.28)	9.89 (3.70)	10.54 (4.09)	13.61 (4.25)	4 > 3 > 2 > 1
Zone-out	9.62 (3.68)	12.56 (4.20)	13.38 (4.45)	15.25 (4.34)	4 > 3 = 2 > 1
Vivid internal world	10.79 (3.39)	13.78 (3.69)	13.77 (3.60)	16.76 (3.72)	4 > 3 = 2 > 1
**DES-T**	5.73 (8.87)	13.62 (13.13)	16.10 (13.34)	30.78 (18.47)	4 > 3 > 2 > 1
**CTQ-SF**					
Cumulative trauma	0.78 (1.16)	1.35 (1.48)	1.48 (1.56)	2.17 (1.63)	4 > 3 = 2 > 1
*No. of individuals with trauma above cut-off*					
Physical abuse, % (*n*)	8.1% (184)	16.4% (62)	20.6% (46)	29.2% (21)	4 = 3 = 2 > 1
Emotional abuse, % (*n)*	12.9% (294)	28.8% (109)	30.5% (68)	44.4% (32)	4 = 3 = 2 > 1
Sexual abuse, % (*n*)	5.3% (120)	11.6% (44)	12.6% (28)	23.6% (17)	4 > 2 > 1; 3 > 1
Physical neglect, % (*n*)	26.0% (593)	39.3% (149)	42.2% (94)	65.3% (47)	4 > 3 = 2 > 1
Emotional neglect, % (*n*)	26.2% (599)	38.8% (147)	41.7% (93)	54.2% (39)	4 = 3 = 2 > 1

Note. CAPE-P15 = Community Assessment of Psychic Experiences-Positive Scale; CTQ-SF = Childhood Trauma Questionnaire-Short Form; DEMO = Dissociative Experience Measures Oxford; DES-T = Dissociative Experiences Scale-Taxon

**Fig. 1:**
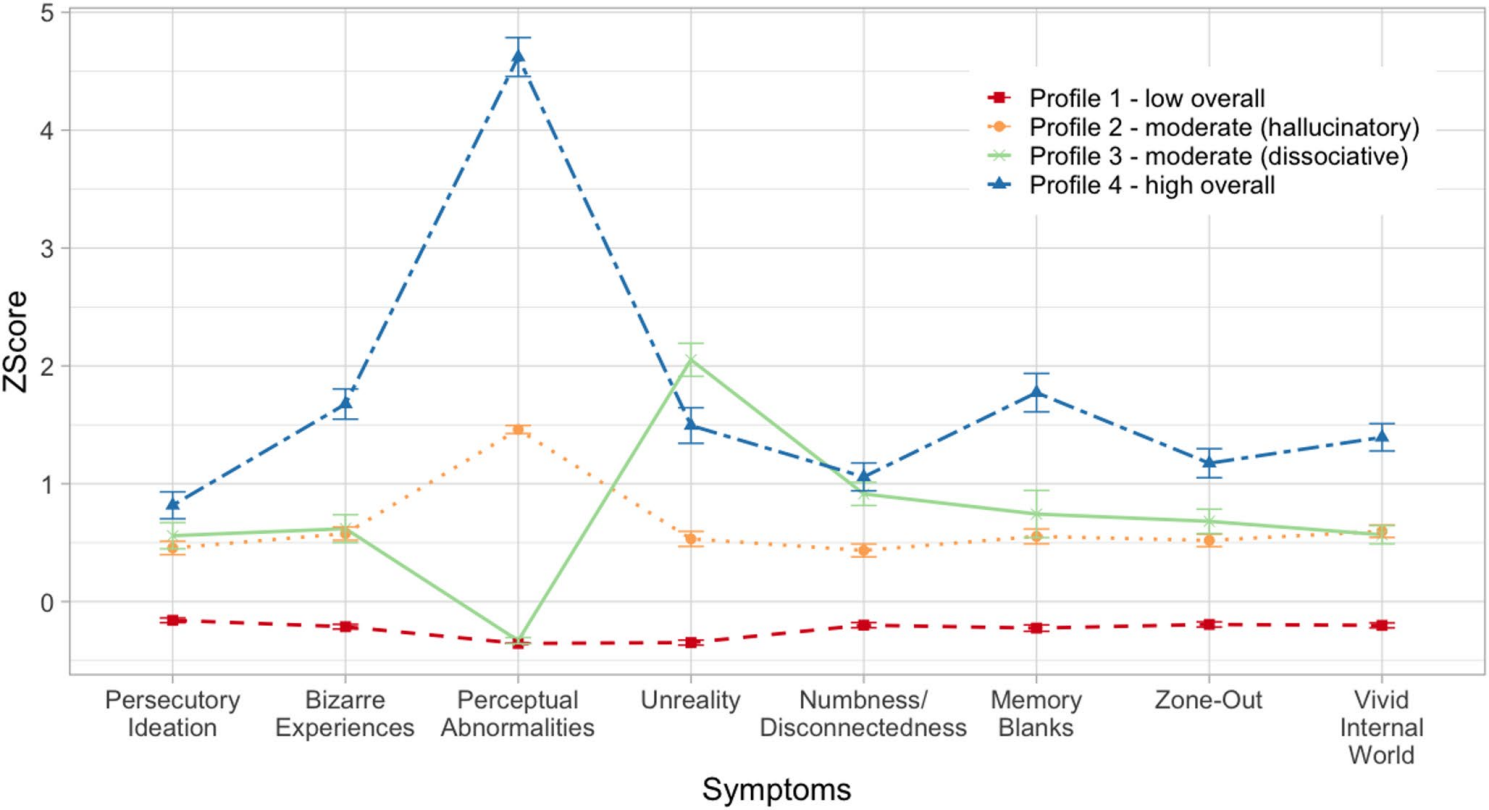
Z-score and 95% confidence interval of positive psychotic and dissociative symptoms for the four profiles

Demographic characteristics and symptom levels for the four profiles are shown in Table 2; Fig. 1. MANOVA revealed significant differences in positive psychotic symptoms (*p*s < 0.001, *ηp2* = 0.09–0.90) and dissociative symptoms (*p*s < 0.001, *ηp2* = 0.14–0.53) across the four profiles. Profile 1 scored the lowest on all CAPE-P15 and DEMO subscores (*p*s < 0.001). The average CAPE-P15 and DEMO subscores of Profiles 2–3 indicated a middle range of severity. On CAPE-P15, Profiles 2 and 3 did not differ in ‘persecutory ideation’ and ‘bizarre experiences’ (*ps* > 0.050); Profile 2 scored higher on ‘perceptual abnormalities’ than Profile 3 (*p* <.001), whose level was not different from that of Profile 1 (*p* >.050). On DEMO, Profile 3 scored higher than Profile 2 on ‘unreality’, ‘numbness/disconnectedness’, and ‘memory blanks’ (*ps* <.050), but not other subscales. Lastly, Profile 4 scored the highest on all CAPE-P15 subscales (*ps* <.001) and most DEMO subscores (*ps* <.001).

The group differences in the proportions of individuals who met the CAPE-P15 cut-off for risk of psychosis [40] were significant, χ^2^(3) = 525.62, *p* <.001, [Profile 1 (*n* = 477; 20.9%), Profile 2 (*n* = 250; 66.0%), Profile 3 (*n* = 122; 54.7%), Profile 4 (*n* = 69; 95.8%)]. The group differences in the proportions of individuals who met the DES-T cut-off for potential dissociative disorder [53] were also significant, χ^2^(3) = 442.55, *p* <.001, [Profile 1 (*n =* 153; 6.7%), Profile 2 (*n =* 97; 25.6%), Profile 3 (*n =* 69; 30.9%), Profile 4 (*n =* 53; 73.6%)]. Additional post-hoc comparisons between Profiles 2 and 3 showed that Profile 2 scored higher on CAPE-P15 total score, whereas Profile 3 scored higher on the DES-T. Accordingly, Profile 1 was labelled as ‘low overall’, Profile 2 as ‘moderate (hallucinatory)’, Profile 3 as ‘moderate (dissociative)’ and Profile 4 as ‘high overall’.

### Prediction of profiles by childhood trauma

**Table 3:** Test of childhood trauma as predictors of latent profiles using multinomial logistic regression

	Adjusted OR (95%CI)	*p*-value
Cumulative		
Profile 2 vs. 1	**1.41 (1.30–1.52)**	**< 0.001**
Profile 3 vs. 1	**1.56 (1.40–1.74)**	**< 0.001**
Profile 4 vs. 1	**1.97 (1.70–2.27)**	**< 0.001**
Profile 3 vs. 2	1.11 (0.99–1.25)	0.101
Profile 4 vs. 2	**1.40 (1.20–1.63)**	**< 0.001**
Profile 4 vs. 3	**1.26 (1.07–1.49)**	**0.015**
**Physical Abuse**		
Profile 2 vs. 1	1.16 (0.80–1.68)	0.470
Profile 3 vs. 1	1.54 (0.97–2.45)	0.140
Profile 4 vs. 1	1.26 (0.63–2.52)	0.554
Profile 3 vs. 2	1.33 (0.79–2.23)	0.347
Profile 4 vs. 2	1.09 (0.53–2.25)	0.822
Profile 4 vs. 3	0.82 (0.38–1.76)	0.574
**Emotional Abuse**		
Profile 2 vs. 1	**2.16 (1.57–2.97)**	**0.001**
Profile 3 vs. 1	**1.90 (1.23–2.92)**	**0.031**
Profile 4 vs. 1	2.86 (1.46–5.63)	0.060
Profile 3 vs. 2	0.88 (0.55–1.42)	0.571
Profile 4 vs. 2	1.33 (0.65–2.70)	0.500
Profile 4 vs. 3	1.51 (0.71–3.22)	0.383
**Sexual Abuse**		
Profile 2 vs. 1	**1.81 (1.22–2.67)**	**0.026**
Profile 3 vs. 1	1.93 (1.15–3.24)	0.068
Profile 4 vs. 1	**3.01 (1.56–5.71)**	**0.041**
Profile 3 vs. 2	1.07 (0.61–1.86)	0.819
Profile 4 vs. 2	1.67 (0.85–3.28)	0.247
Profile 4 vs. 3	1.56 (0.75–3.24)	0.337
**Physical Neglect**		
Profile 2 vs. 1	1.39 (1.04–1.85)	0.056
Profile 3 vs. 1	1.78 (1.13–2.81)	0.059
Profile 4 vs. 1	**3.59 (1.99–6.48)**	**0.017**
Profile 3 vs. 2	1.28 (0.78–2.11)	0.382
Profile 4 vs. 2	2.59 (1.34–4.87)	0.057
Profile 4 vs. 3	2.02 (0.99–4.10)	0.164
**Emotional Neglect**		
Profile 2 vs. 1	1.05 (0.77–1.41)	0.774
Profile 3 vs. 1	1.10 (0.69–1.78)	0.699
Profile 4 vs. 1	0.95 (0.51–1.74)	0.852
Profile 3 vs. 2	1.06 (0.63–1.78)	0.844
Profile 4 vs. 2	0.90 (0.47–1.73)	0.748
Profile 4 vs. 3	0.86 (0.41–1.78)	0.653

Note. Odd ratios are adjusted for age, gender, and effects of other childhood trauma subtypes. Statistically significant coefficients are in bold typeface

Results of multinomial logistic regression of latent profiles on childhood trauma at baseline are shown in Table 3. ANOVA revealed significant differences in cumulative childhood trauma (*p* <.001, *ηp2* = 0.06) across profiles, with Profile 4 reporting the highest count, followed by Profiles 2 and 3, which did not differ from each other (*p* >. 050), and then Profile 1 (*ps* <. 001). Multinomial logistic regression indicated that experiencing one additional trauma increased the odds of being in Profiles 2–4 when compared to Profile 1 (*ORs*: 1.41–1.97, *ps* < 0.001), and being in Profile 4 as opposed to Profiles 2 and 3 (*ORs*: 1.26–1.40, *ps* < 0.050).

With Profile 1 as reference group, presence of emotional abuse increased the odds of being in Profiles 2 and 3 (*ORs*: 1.90–2.16, *ps* < 0.050); presence of sexual abuse increased the odds of being in Profiles 2 and 4 (*ORs*: 1.81–3.01, *ps* < 0.050); presence of physical neglect increased the odds of being in Profile 4 (*OR*: 3.59, *p* =.017). No particular subtypes of abuse or neglect differentiated between Profiles 2–4 (*ps* > 0.050). The effects shown were adjusted for age, gender, and other childhood trauma subtypes.

### Emotional and functional outcome at baseline and follow-up

**Table 4:** Group comparisons on emotional and functional outcomes at baseline and follow-up

M (SD)	Profile 1	Profile 2	Profile 3	Profile 4	Overall test	Pairwise comparison (Bonferroni)
*Baseline*						
PHQ-9	5.15 (4.16)	7.68 (5.00)	8.32 (5.07)	10.68 (5.14)	χ^2^(3) = 227.61, *p* <.001	4 > 3 > 2 > 1
GAD-7	5.02 (4.06)	6.84 (4.34)	7.33 (4.37)	8.78 (4.20)	χ^2^(3) = 151.90, *p* <.001	4 = 3 > 2 > 1
WHODAS 2.0	4.60 (4.91)	7.31 (6.12)	7.57 (6.13)	11.57 (7.65)	χ^2^(3) = 161.86, *p* <.001	4 > 3 = 2 > 1
*Follow*-up						
PHQ-9	5.09 (4.14)	6.99 (4.80)	7.80 (5.06)	8.57 (4.51)	χ^2^(3) = 104.07, *p* <.001	4 = 3 > 2 > 1
GAD-7	5.01 (4.02)	6.79 (4.39)	7.16 (4.80)	7.11 (3.93)	χ^2^(3) = 78.85, *p* <.001	4 = 3 = 2 > 1
WHODAS 2.0	4.76 (4.99)	7.30 (6.44)	7.07 (5.89)	9.46 (7.63)	χ^2^(3) = 77.00, *p* <.001	4 = 3 = 2 > 1

Note. GAD-7 = Generalised Anxiety Disorder 7-item Scale; PHQ-9 = Patient Health Questionnaire-9; WHODAS 2.0 = World Health Organisation Disability Assessment Schedule 2.0

Means and SDs of emotional and functional outcomes at baseline and follow-up are shown in Table 4. At baseline, Profile 4 had the worst outcome overall, with the highest PHQ-9, GAD-7, and WHODAS 2.0 scores across all profiles (*p*s < 0.01), except a GAD-7 score not significantly different from Profile 3 (*p =*.052). Profile 3 had higher PHQ-9 and GAD-7 scores than Profile 2 (*p*s < 0.050), but they did not differ on WHODAS 2.0 (*p* =.257). Profile 1 scored lower on every pairwise comparison of all measures (*p*s < 0.001).

At follow-up, Profiles 3 and 4 had comparable PHQ-9 scores (*p* =.611), both scoring higher than Profile 2 (*ps* < 0.050), followed by Profile 1 (*p*s < 0.001). On GAD-7 and WHODAS 2.0, Profiles 2–4 scored higher than Profile 1 (*ps* < 0.001), with no significant differences amongst Profiles 2–4 (*ps* > 0.050).

## Discussion

The current study examined the distinct patterns of co-occurrence of positive psychotic and dissociative symptoms in a large community sample of adults. Latent profile analysis of subscale-level symptoms yielded a four-profile solution. Most participants (Profile 1; 77.2%) showed consistently low levels of both symptom clusters. Two intermediate profiles captured differential patterns of expression: Profile 2 (12.8%) was characterised primarily by increased hallucinations, while Profile 3 (7.5%) showed heightened depersonalisation/derealisation (i.e., DEMO subscale of unreality), numbness, and memory blanks. A small group (Profile 4; 2.4%) showed high severity across both symptom clusters concurrently that could be at risk for comorbid psychotic and dissociative disorders.

Consistent with prior work indicating most individuals in the general population experience only mild or transient symptoms [8, 54], most participants showed low symptom levels; nevertheless, 22.8% fell into three subgroups with moderate-to-high positive psychotic and dissociative symptoms. The profiles reveal heterogeneity in co‑occurrence patterns. For instance, while dissociation – particularly depersonalisation/derealisation (DD) – is recognised as associated with hallucinations [26], our results showed that they only co-occur in Profiles 2 and 4, but not Profile 3, whose hallucination level was non-distinguishable from Profile 1. This pattern suggests that individuals with heightened DD do not necessarily develop hallucinations, but those with heightened hallucinations are more frequently accompanied by DD, a finding compatible with DD contributing to the aetiology of hallucinations [55]. The identification of these latent subgroups in the general population supports multidimensional screening and early detection in preclinical or prodromal stages – with particular attention to dissociative symptoms, which are often under‑recognised [56].

Our results indicated that the moderate-to-high profiles present a pattern of risk factors akin to those found in patients with psychotic or dissociative disorders (e.g [33, 57]). Aligning with findings of dose-response relationship [30, 31], exposure to additional childhood trauma was linked to profiles with higher symptom severity. Specificity between several trauma subtypes and profiles were also observed. Emotional abuse distinguished Profiles 2 and 3 from Profile 1, suggesting it as a risk factor for both moderate positive psychotic and dissociative symptoms. Sexual abuse consistently predicted subgroups with heightened hallucinations (Profiles 2 and 4), corroborating its pronounced effect on predisposition to psychosis, particularly hallucinations [32], through the mediating role of dissociation [57].

Profiles characterised by elevated positive psychotic and dissociative symptoms also exhibited persistent distress. Notably, subgroups characterised by prominent dissociative symptoms (Profiles 3 and 4) consistently exhibited higher levels of depression compared to other profiles at both baseline and follow-up. Similarly, Maaranen et al. [11] found that stable or new cases of high dissociation over a 3-year period were associated with increased depression severity, whereas recovery from high dissociation was related to a decrease in depression severity. While causality between dissociation and depression was not directly established in Maaranen et al. [11] and the current study, previous research [58, 59] showed that psychological treatments targeting dissociation effectively reduced depression severity, indicating that heightened dissociative symptoms may entail depression or mood problems. Increased anxiety was also shared among the moderate-to-high subgroups. However, the relationship between anxiety and positive psychotic as well as dissociative symptoms could be dynamic and reciprocal, as previous findings suggested that anxiety may drive these symptoms, which in turn reinforce anxiety [60, 61]. Our results supported that community-dwelling individuals who experience co-occurring positive psychotic and dissociative symptoms may be prone to a wide range of psychiatric symptoms as well.

Regarding functional impairment, the moderate-to-high subgroups reported impairment scores two to three times higher than the general population norm [46] at both timepoints. Altogether, the findings on the outcome measures underscore the detrimental impact of subclinical positive psychotic and dissociative symptoms on one’s mental well-being and global functioning abilities [11, 18], even in the absence of diagnosed psychotic or dissociative disorders. Timely recognition of these symptoms is pivotal for preventing progression into clinical conditions and, equally importantly, for enhancing mood and functioning in these specific groups as soon as symptoms emerge.

One caveat of the current study is the inclusion of only a single follow-up. Repeated assessment at two time points may at best provide a proof-of-concept for changes, but may not be adequate in capturing meaningful developmental trajectories of symptomatology. Therefore, we have refrained from interpreting changes in anxiety, depression, and functioning using this sample. Prospective clinical outcomes such as transition to a psychotic or dissociative disorder, or development of other psychiatric diagnoses, also cannot be predicted. Another limitation is the predominantly female and relatively well-educated sample, which may restrict the generalisability of the findings. Replication in a more diverse sample (e.g., gender-balanced, less educated, social minorities) is needed. Lastly, reliance on self-report measures may introduce bias. For example, Chiu et al. [62] found that self-reported levels of positive psychotic symptoms in mood disordered patients (who experience dissociation) can be even higher than actively psychotic patients with schizophrenia, especially when the respondents are in a mood episode. Although validity checks were implemented, self-reports remain subject to participants’ interpretations. Future investigations would benefit from incorporating interviewer-rated measures to better validate symptom severity.

## Conclusions

This study identified distinct subgroups in the general population characterised by differing patterns of co-occurring positive psychotic and dissociative symptoms, which are meaningfully associated with exposure to childhood trauma. Although the symptoms are subclinical, their co-occurrence is associated with poorer emotional and functional outcomes, rendering early interventions necessary to relieve these individuals from distress and prevent further deterioration in functioning. Replication and longitudinal examination of these profiles over multiple waves of measurements would further inform their developmental trajectories.

## Supplementary Information

Below is the link to the electronic supplementary material.

Supplementary Material 1.

## Data Availability

Data is available from the corresponding author upon request.
