# Supplementary material for: A latent profile analysis of positive psychotic symptoms and dissociative symptoms in the general population: their associations with childhood trauma and outcomes
Source: Soc Psychiatry Psychiatr Epidemiol. 2025 Sep 15;61(2):311–20. doi: 10.1007/s00127-025-02992-3 (PMC12948800; doi:10.1007/s00127-025-02992-3)
Supplement: Supplementary file 1 — Supplementary Material 1. [file 127_2025_2992_MOESM1_ESM.docx]

A latent profile analysis of positive psychotic symptoms and dissociative symptoms in the general population: Their associations with childhood trauma and outcomes

Supplemental materials

**Supplemental Table 1**

*Characteristics of the sample at baseline (N = 2,958)*

|  | Mean (SD) / *n* (%) |  |
| --- | --- | --- |
| **Age** | 34.69 (12.45) |  |
| **Gender** |  |  |
| Female | 2146 (72.5%) |  |
| Male | 812 (27.5%) |  |
| **Ethnicity** |  |  |
| Chinese | 2864 (96.8%) |  |
| Eastern South and South Asian | 39 (1.3%) |  |
| Others (unspecified) | 55 (1.9%) |  |
| **Educational attainment** |  |  |
| Secondary or below | 883 (29.9%) |  |
| Associate degree or higher diploma | 278 (9.4%) |  |
| Bachelor’s degree | 1087 (36.7%) |  |
| Master’s degree or above | 710 (24.0%) |  |
| **Employment status** |  |  |
| Full-time employment | 1696 (57.3%) |  |
| Part-time employment (full-time student) | 645 (21.8%) |  |
| Part-time employment (not full-time student) | 183 (6.2%) |  |
| Not working and searching for job | 136 (4.6%) |  |
| Not working and not searching for job | 298 (10.1%) |  |
| **Monthly household income** |  |  |
| < HKD 10,000 | 211 (7.1%) |  |
| HKD 10,000–29,999 | 904 (30.6%) |  |
| HKD 30,000–49,999 | 870 (29.4%) |  |
| HKD 50,000–99,999 | 743 (25.1%) |  |
| > HKD 100,000 | 230 (7.8%) |  |
|  | Mean (SD) | Cronbach’s alpha |
| **Positive psychotic symptoms** |  |  |
| CAPE-P15 frequency total | 21.27 (4.35) | 0.81 |
| Persecutory ideation | 8.37 (2.12) | 0.72 |
| Bizarre experiences | 9.65 (2.54) | 0.72 |
| Perceptual abnormalities | 3.25 (0.71) | 0.66 |
| **Dissociative symptoms** |  |  |
| DEMO total | 49.80 (14.88) | 0.94 |
| Unreality | 8.61 (3.54) | 0.89 |
| Numbness/disconnectedness | 11.02 (4.66) | 0.88 |
| Memory blanks | 8.21 (3.05) | 0.85 |
| Zone-out | 10.42 (4.12) | 0.89 |
| Vivid internal world | 11.54 (3.74) | 0.80 |
| DES-T | 8.13 (11.40) | 0.88 |
| **Childhood trauma** |  |  |
| Total score | 40.07 (13.16) | 0.91 |
| Physical abuse | 6.39 (2.52) | 0.84 |
| Emotional abuse | 9.13 (3.75) | 0.82 |
| Sexual abuse | 5.49 (1.51) | 0.83 |
| Physical neglect | 8.13 (3.56) | 0.72 |
| Emotional neglect | 10.93 (5.56) | 0.90 |
| **PHQ-9** | 5.85 (4.58) | 0.86 |
| **GAD-7** | 5.52 (4.230 | 0.91 |
| **WHODAS 2.0** | 5.34 (5.47) | 0.87 |

*Note*. CAPE-P15 = Community Assessment of Psychic Experiences-Positive Scale; CTQ-SF = Childhood Trauma Questionnaire-Short Form; DEMO = Dissociative Experience Measures Oxford; DES-T = Dissociative Experience Scale-Taxon; GAD-7 = Generalised Anxiety Disorder 7-item Scale; PHQ-9 = Patient Health Questionnaire-9; WHODAS 2.0 = World Health Organisation Disability Assessment Schedule 2.0

**Supplemental Table 2**

*Spearman's rho Correlation between Indicator Variables at Baseline and Follow-up*

|  |  | Baseline | | | | | | | | 6-month | | | | | | | |
| --- | --- | --- | --- | --- | --- | --- | --- | --- | --- | --- | --- | --- | --- | --- | --- | --- | --- |
|  |  | 1 | 2 | 3 | 4 | 5 | 6 | 7 | 8 | 1 | 2 | 3 | 4 | 5 | 6 | 7 | 8 |
| 1. | Persecutory ideation | -- |  |  |  |  |  |  |  | 0.61 | 0.42 | 0.19 | 0.33 | 0.41 | 0.26 | 0.37 | 0.37 |
| 2. | Bizarre experiences | 0.52 | -- |  |  |  |  |  |  | 0.38 | 0.62 | 0.21 | 0.42 | 0.42 | 0.37 | 0.43 | 0.42 |
| 3. | Perceptual Abnormalities | 0.23 | 0.30 | -- |  |  |  |  |  | 0.18 | 0.25 | 0.49 | 0.23 | 0.19 | 0.21 | 0.21 | 0.23 |
| 4. | Unreality | 0.41 | 0.51 | 0.30 | -- |  |  |  |  | 0.33 | 0.44 | 0.27 | 0.60 | 0.45 | 0.33 | 0.42 | 0.40 |
| 5. | Numbness/ disconnectedness | 0.48 | 0.50 | 0.24 | 0.57 | -- |  |  |  | 0.41 | 0.46 | 0.19 | 0.47 | 0.68 | 0.35 | 0.50 | 0.42 |
| 6. | Memory blanks | 0.33 | 0.45 | 0.28 | 0.46 | 0.46 | -- |  |  | 0.27 | 0.40 | 0.23 | 0.38 | 0.37 | 0.57 | 0.44 | 0.38 |
| 7. | Zone-out | 0.39 | 0.50 | 0.27 | 0.49 | 0.58 | 0.57 | -- |  | 0.33 | 0.44 | 0.19 | 0.42 | 0.50 | 0.42 | 0.64 | 0.42 |
| 8. | Vivid internal world | 0.43 | 0.52 | 0.30 | 0.47 | 0.49 | 0.45 | 0.53 | -- | 0.36 | 0.42 | 0.22 | 0.39 | 0.39 | 0.34 | 0.40 | 0.56 |

*Note.* All correlations are significant at *p <* 0.001.
